# A *Coxiella burnetii* phospholipase A homolog *pldA* is required for optimal growth in macrophages and developmental form lipid remodeling

**DOI:** 10.1186/s12866-018-1181-0

**Published:** 2018-04-16

**Authors:** Christopher M. Stead, Diane C. Cockrell, Paul A. Beare, Heather E. Miller, Robert A. Heinzen

**Affiliations:** 1 Coxiella Pathogenesis Section, Laboratory of Bacteriology, Rocky Mountain Laboratories, National Institute of Allergy and Infectious Diseases, National Institutes of Health, Hamilton, Montana USA; 2 Department of Chemistry, New Mexico Highlands University, Las Vegas, New Mexico USA

**Keywords:** *Coxiella*, Lipid, Fatty acids, Phospholipase A, Small cell variant

## Abstract

**Background:**

Many gram-negative bacteria produce an outer membrane phospholipase A (PldA) that plays an important role in outer membrane function and is associated with virulence.

**Results:**

In the current study, we characterized a *pldA* mutant of *Coxiella burnetii,* an intracellular gram-negative pathogen and the agent of human Q fever. The *C. burnetti pldA* open reading frame directs synthesis of a protein with conserved PldA active site residues. A *C. burnetii* Δ*pldA* deletion mutant had a significant growth defect in THP-1 macrophages, but not axenic medium, that was rescued by complementation. Thin layer chromatography was employed to assess whether *pldA* plays a role in remodeling membrane lipids during *C. burnetii* morphological differentiation. Extracted lipids were analyzed from replicating, logarithmic phase large cell variants (LCVs), non-replicating, stationary phase small cell variants (SCVs), and a mixture of LCVs and SCVs. Similar to *Escherichia coli*, all three forms contained cardiolipin (CL), phosphatidylglycerol (PG) and phosphatidylethanolamine (PE). However, PE and PG were present in lower quantities in the SCV while three additional lipid species were present in higher quantities. Co-migration with standards tentatively identified two of the three SCV-enriched lipids as lyso-phosphatidylethanolamine, a breakdown product of PE, and free fatty acids, which are generally toxic to bacteria. Developmental form lipid modifications required the activity of PldA.

**Conclusions:**

Collectively, these results indicate developmentally-regulated lipid synthesis by *C. burnetii* contributes to colonization of macrophages and may contribute to the environmental stability and the distinct biological properties of the SCV.

**Electronic supplementary material:**

The online version of this article (10.1186/s12866-018-1181-0) contains supplementary material, which is available to authorized users.

## Background

*Coxiella burnetii* is a gram-negative intracellular pathogen noted for high environmental stability and a low infectious dose via the aerosol route of infection [1]. *C. burnetii* causes an acute flu-like illness known as Q fever. Following infection, the organism traffics to a vacuole with lysosomal characteristics [2]. Replication of the organism proceeds via a bi-phasic developmental cycle, during which it transitions from a large cell variant (LCV) to a small cell variant (SCV) developmental form [3–5]. The LCV is considered the replicative form and is present during logarithmic growth. As bacterial growth enters stationary phase, LCVs differentiate into SCVs. As compared to LCVs, SCVs have low metabolic activity and increased resistance to osmotic and physical stressors [5]. These resistance properties are thought to promote environmental stability by the SCV [4].

Despite the apparent importance of SCVs in *C. burnetii* disease transmission and pathogenesis, relatively little is known about biochemical changes during transition that confer the unique biological properties of the SCV. Ultrastructural differences between LCVs and SCVs predicted to promote SCV stability include a thicker cell envelope, different peptidoglycan cross linking, condensed chromatin, and synthesis of two highly basic DNA binding proteins [4–6].

The gram-negative cell envelope is composed of an inner and outer membrane, with peptidoglycan separating the two membranes. Both leaflets of the inner membrane are composed of phospholipids, whereas, the outer membrane has an inner leaflet of phospholipids and an outer leaflet of lipopolysaccharide. Phospholipid modifications are common in bacteria and can provide an increase in resistance properties [7]. For example, cyclopropanation of phospholipid acyl chains in *Escherichia coli* increases resistance to acid stress [8].

Considering the importance of phospholipids in cell envelope function, few studies of these molecules have been conducted in *C. burnetii*. A report published in 2002 demonstrated phosphatidylethanolamine (PE), phosphatidylserine (PS), phosphatidylcholine (PC), and phosphatidylglycerol (PG) in both virulent phase I and avirulent phase II *C. burnetii,* which produce full-length and truncated lipopolysaccharide, respectively [9]. Phosphatidylinositol (PI) was also detected in phase II bacteria [9]. A subsequent study showed no PI in *C. burnetii,* which is consistent with the absence of genes responsible for synthesis of this lipid [10, 11]. Moreover, PI is common in eukaryotes but rare in eubacteria [12]. Both studies used *C. burnetii* purified from infected hen’s eggs; thus, it is possible that contaminating eukaryotic host cell membrane was in bacterial preparations. The majority of *C. burnetii* fatty acids are branched, with little difference in the profiles between the cell envelope of LCVs and SCVs [13, 14].

In the current study, we utilized axenic culture to characterize the lipid profile of *C. burnetii* as it transitions from the LCV to SCV developmental form. We show marked changes in lipid content during differentiation that are attributable to the activity of a predicted outer membrane phospholipase A (PldA).

## Results

### *C. burnetii pldA* is required for optimal growth in macrophages

**Fig. 1:**
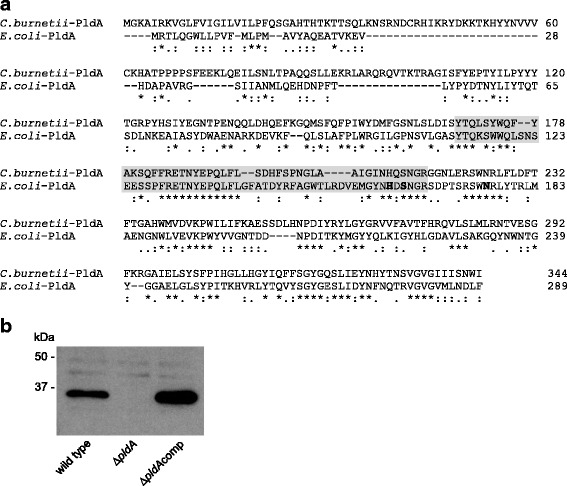
Generation of a *C. burnetii pldA* mutant. **a** Alignment of PldA from *E. coli* and *C. burnetii*. The grey region shows the consensus sequence motif YTQ-X_n_-G-X_2_-H-X-SNG for PldA enzymes. Bold amino acids show known active site residues [15]. **b** Lysates from *C. burnetii* wild type, Δ*pldA,* and Δ*pldA*comp strains after 6 days of growth in ACCM-2 (early SCV) were probed by immunoblotting with an anti-PldA polyclonal antibody

CBU0489 is annotated as an outer membrane phospholipase A (*pldA*)-encoding gene [11]. *C. burnetii* PldA displayed 40% amino acid identity (E-value of 1 × 10^− 44^) with the *E. coli* homolog (accession no. BAE77480.1). Amino acid residues comprising the PldA consensus motif and active site residues were highly conserved [15] (Fig. 1a). To investigate the role of *pldA* in *C. burnetii* lipid metabolism, a null mutant was generated using allelic exchange (Additional file 1: Figure S1). The mutant was subsequently complemented using a Tn7 construct to insert a single copy of *pldA* into the chromosome under the control of a native promoter [16]. An immunoblot of cell lysates prepared from wild type, Δ*pldA,* and Δ*pldA*comp *C. burnetii* strains demonstrated loss of PldA in the mutant, which was restored upon complementation (Fig. 1b).

**Fig. 2:**
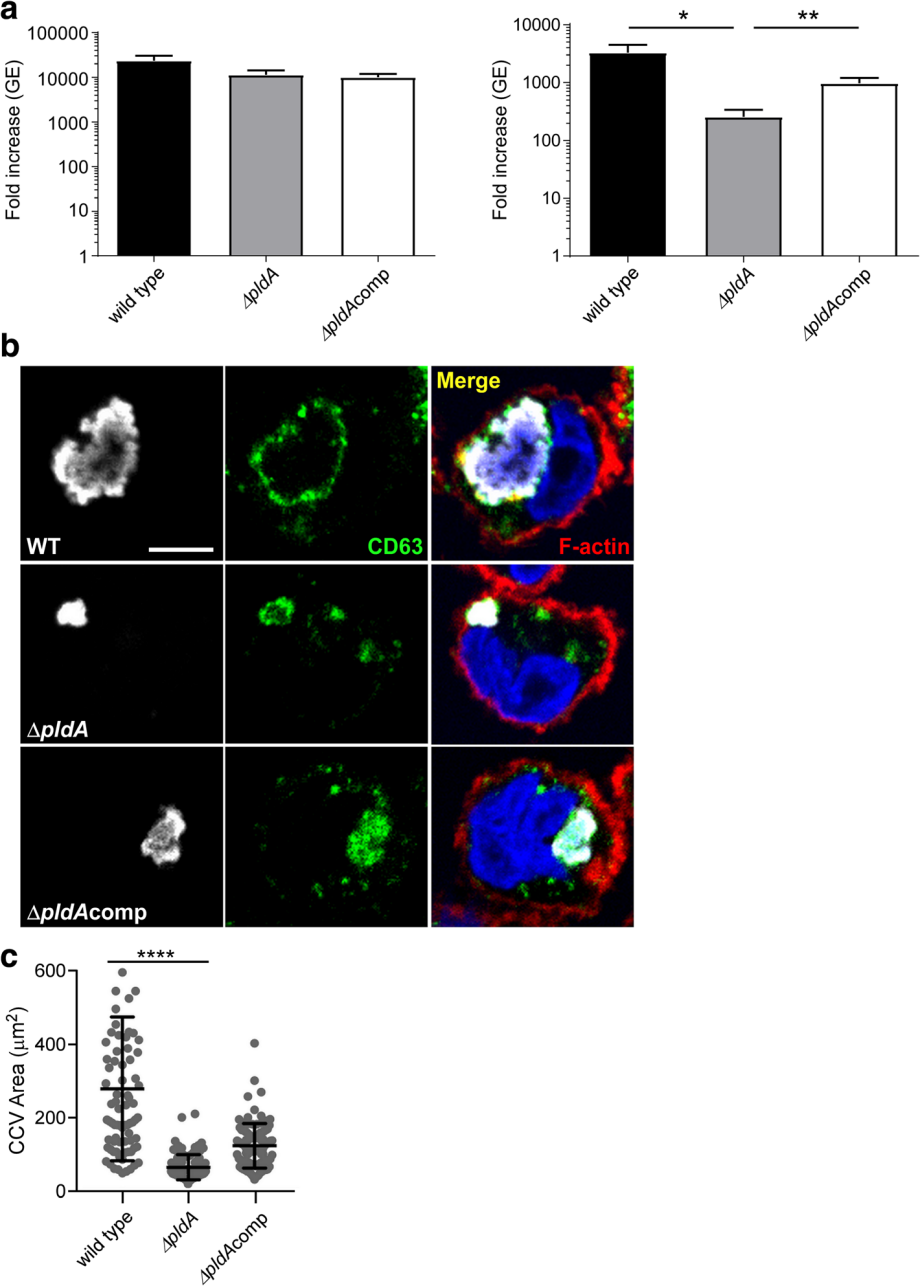
A Δ*pldA* mutant has impaired growth in THP-1 macrophages. **a** Growth of *C. burnetii* wild type, Δ*pldA,* and Δ*pldA*comp strains in ACCM-2 (left panel) and THP-1 macrophages (right panel). Data represent fold increases in genome equivalents (GE) after 6 days of growth (early SCV) for 3 independent experiments performed in triplicate. Asterisks indicate a statistically significant difference (* = *P* < 0.05, ** = *P* < 0.01) as determined by the unpaired Student *t* test. **b** Immunofluorecence micrographs of *C. burnetii* wild type, Δ*pldA,* and Δ*pldA*comp strains after 3 days of growth (LCV). Bacteria are colored white, the vacuole membrane green, the THP-1 cell border red, and the nucleus blue. **c** Size of *Coxiella*-containing vacuoles (CCV) at 3 days post-infection, Vacuole size was measured with Fiji and the data are representative of three independent experiments. Asterisks indicate a statistically significant difference. (**** = P < 0.0001*).* Scale bar, 5 μm

To determine the importance of *pldA* during host cell infection, growth of the Δ*pldA* and Δ*pldA*comp strains were assessed in synthetic media and THP-1 macrophages. When axenically cultivated, mutant and wild type bacteria replicated to similar levels (Fig. 2a). However, during infection of macrophages, mutant bacteria displayed a significant growth defect that was partially restored upon complementation (Fig. 2a). The mutant phenotype correlated with significantly smaller *Coxiella*-containing vacuoles (CCV) with fewer organisms (Fig. 2b and c).

### *C. burnetii* produces a unique lipid profile that changes during the LCV to SCV transition

**Fig. 3:**
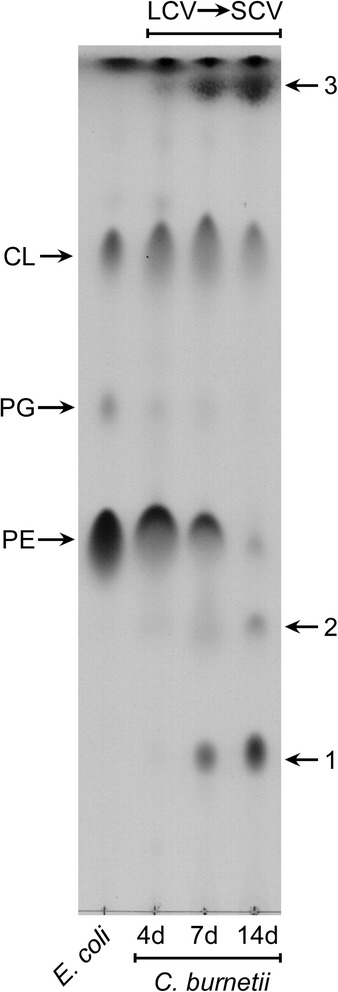
The *C. burnetii* lipid profile changes during LCV to SCV transition. Lipids were isolated from 4, 7, and 14 day *C. burnetii* axenic cultures to represent different stages of the developmental cycle. Lipids were analyzed by TLC and spotted according to mass: *C. burnetii* = 550 μg, *E. coli* = 200 μg. Lipid species 1, 2, and 3 were enriched in the SCV while phosphatidylethanolamine and phosphatidylglycerol were diminished. Abbreviations: CL, cardiolipin; PG, phosphatidylglycerol; PE, phosphatidylethanolamine

To gain insight into *pldA*-driven lipid modifications associated with the development cycle of *C. burnetii*, we first examined the phospholipid content of wild type bacteria. To avoid contamination of host cell-derived lipids, *C. burnetii* lipid analysis was conducted using bacteria cultured in ACCM-2 (acidified citrate cysteine medium-2), which is an appropriate model for studying *C. burnetii* developmental transitions [6]. Four, 7, and 14 day time points were taken to represent LCV, LCV + SCV, and SCV developmental forms, respectively. Lipids were isolated as described in the Materials and Methods and analyzed by thin layer chromatography (TLC) along with *E. coli* lipids. *E. coli* is considered a model organism for gram-negative phospholipid biosynthesis [17, 18]. TLC showed that the *C. burnetii* lipid profile is dynamic during LCV to SCV transition and different from the lipid profile of *E. coli* (Fig. 3). Both *E. coli* and *C. burnetii* synthesized three major lipid species based on retardation factor (R_f_): CL (R_f_ = 0.77), PG (R_f_ = 0.59), and PE (R_f_ = 0.43) (Fig. 3) [19]. However, *C. burnetii* produced three additional lipid species that were substantially increased in SCVs. In contrast, PG and PE were reduced in SCVs.

### *C. burnetii* likely produces lyso-phosphatidylethanolamine and free fatty acids

**Fig. 4:**
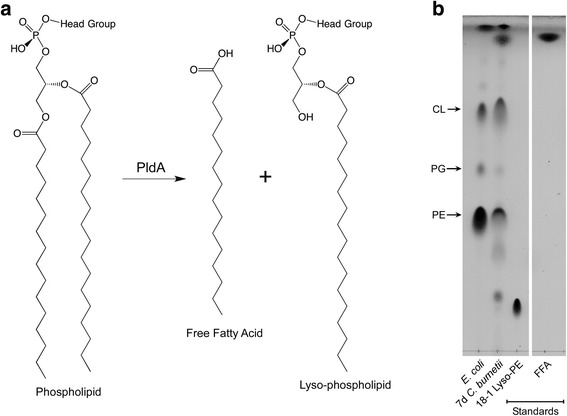
Identification of *C. burnetii* SCV enriched lipids. PldA hydrolyzes a variety of phospholipids that contain a polar head group and acyl-chains containing 14 or more carbons at the *sn*-1 or *sn*-2 position [20, 21]. **a** Hydrolysis of a phospholipid acyl chain at the *sn*-1 position to produce a lyso-phospholipid and a free fatty acid. **b** R_f_ values were compared to purchased standards. All samples were run on the same TLC plate with the image split to exclude irrelevant samples. Abbreviations: PE; phosphatidylethanolamine; PG, phosphatidylglycerol; CL, cardiolipin; FFA, free fatty acids; 18–1 Lyso-PE, 18–1 lyso-phosphatidylethanolamine

PldA catalyzes the removal of an acyl chain from a phospholipid to produce a lyso-phospholipid (lyso-PL) and a free fatty acid (FFA) (Fig. 4a) [20, 21]. Therefore, it is logical to assume that some of the lipids enriched in the SCV could be Lyso-PLs or FFAs. To investigate this assumption, an 18–1 lyso-phosphatidylethanolamine (18–1 Lyso-PE) and palmitoleic acid standard were analyzed alongside *C. burnetii* lipids. By TLC, an 18–1 Lyso-PE standard (R_f_ = 0.14) had a similar R_f_ to lipid 1 (R_f_ = 0.16), putatively identifying this species as Lyso-PE (Fig. 4b). Lipid 3 (R_f_ = 0.96) had a similar R_f_ to the palmitoleic acid standard (R_f_ = 0.96), putatively identifying this species as FFA.

### *C. burnetii pldA* participates in lipid remodeling associated with LCV to SCV transition

**Fig. 5:**
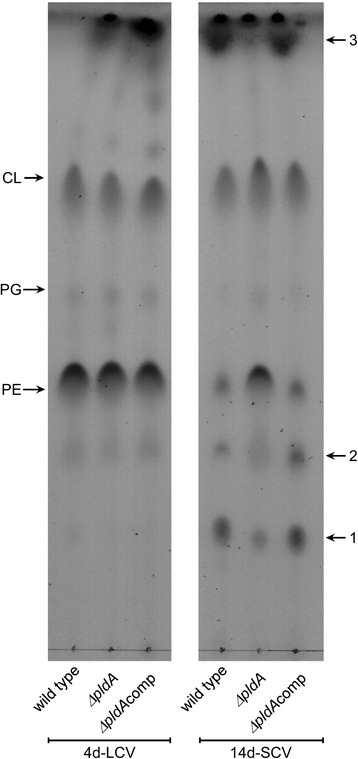
PldA is required for *C. burnetii* lipid changes during developmental transition. LCVs and SCVs were generated by growing *C. burnetii* wild type, Δ*pldA*,and Δ*pldA*comp strains in ACCM-2 for 4 and 14 days, respectively. Lipids were isolated and 400 μg of each sample was analyzed by TLC. Abbreviations: PE, phosphatidylethanolamine; PG, phosphatidylglycerol; CL, cardiolipin

The phospholipid profile of Δ*pldA* and Δ*pldA*comp strains at 4 and 14 days were compared to wild type bacteria (Fig. 5). The three phospholipid profiles at day 4 appeared the same, However, at day 14, the decrease in PE and PG was no longer evident in the Δ*pldA* mutant nor was the increase in lipids 1, 2, and 3. The wild type profile was restored in the Δ*pldA*comp strain. These data indicate *pldA* is responsible for membrane remodeling of the SCV developmental form.

## Discussion

Compositional differences between LCV and SCV developmental forms that contribute to their distinct biological and structural properties are poorly defined. A few proteins have been identified that are differentially synthesized by the LCV and SCV, including the small basic DNA binding proteins ScvA and Hq1 that are associated with the condensed chromatin of SCVs [22–24]. Warrier et al. [25] defined 15 developmentally regulated small RNAs that may play roles in differentiation. Sandoz and coworkers [26] recently demonstrated major changes in peptidoglycan structure during LCV to SCV transition. Here, we demonstrate additional changes to the SCV cell envelope that involve PldA-dependent changes in lipid composition.

PldA-dependent breakdown of PE produces FFA and Lyso-PE. The accumulation of FFA by the *C. burnetii* SCV is perplexing as these molecules are considered toxic to bacteria [27]. In fact, we are aware of only one organism that stores large amounts of medium-to-long chain saturated fatty acids, an anaerobic bacterium known as G12 that is related to *Eubacterium cylidroides* [28]. This bacterium appears to accumulate FFA during homeoviscous adaptation to environmental stress. Many bacterial species utilize stores of lipophilic compounds as energy and carbon sources, generally in the form of polyhydroxyalkanoates, such as poly(3-hydroxybutyrate) [29]. How *C. burnetii* tolerates FFA toxicity and the potential role(s) these compounds play in pathogen physiology are intriguing questions. Given that the greatest amount of FFA is seen in the SCV, these lipid molecules may serve as a nutrient source for outgrowth of the LCV during the initial stages of infection, when the CCV may be limited in nutrients [2, 30].

Lysophopholipids affect membrane stiffness and fluidity, which in turn influences membrane permeability and pore function [20, 31]. PldA-dependent accumulation of lysophopholipids in the *Helicobacter pylori* membrane promotes release of urease and VacA toxin [32, 33]. Both molecules enhance adherence to epithelial cells and development of ulcer disease [32, 33]. In *E. coli,* PldA facilitates release of bacteriocins [34, 35]. *Shigella flexneri* needs PldA for efficient type III secretion and to maintain membrane integrity [36]. We show that PldA-deficient *C. burnetii* clearly has a growth defect in human macrophages, although the precise mechanism of this attenuation remains to be defined. One possibility is that PldA contributes to a recently described Sec-mediated secretion system of *C. burnetii* [37]. Another possibility is that mutant SCVs are less resistant to the lysosomal environment.

## Conclusion

In this work, developmentally-regulated synthesis of *C. burnetii* lipids was described. PldA was responsible for enrichment of Lyso-PE and FFA in the SCV. These data, along with major modifications of SCV peptidoglycan [26], indicate the *C. burnetii* cell envelope undergoes substantial remodeling during morphologic differentiation. PldA also promotes pathogen growth in the harsh, lysosome-like environment of macrophages. Further characterization of PldA and the unusual lipids generated by the enzyme will provide needed insight into *C. burnetii* resistance and pathogenesis.

## Methods

### Bacterial and mammalian cell culture

Bacterial strains are described in Additional file 2: Table S1. ACCM-2 or ACCM-2 agarose was employed to grow *C. burnetii* as previously described [38]. *E. coli* strains were grown in Luria-Bertani (LB) broth at 37 °C. *E. coli* W3110 cells were used for lipid isolation while *E. coli* Stellar cells were used for recombinant DNA procedures. LB agar plates containing 50 μg of kanamycin/ml or 10 μg of chloramphenicol/ml were used to select *E. coli* transformants. The human acute monocytic leukemia cell line THP-1 (TIB-202; American Type Culture Collection) was grown at 37 °C and 5% CO_2_ in RPMI 1640 medium (Invitrogen) supplemented with 10% fetal bovine serum (Hyclone).

### Recombinant DNA techniques

Plasmids used in the study are listed in Additional file 2: Table S1. Accuprime Pfx DNA polymerase (Invitrogen) and oligonucleotide primers (Integrated DNA Technologies) were employed in PCR. Primer sequences are listed in Additional file 3: Table S2. PCR products were cloned using the In-Fusion PCR cloning system (BD Clontech). Restriction enzymes were purchased from New England Biolabs.

### Construction of pJC-amp and pMini-Tn7T-Kan

For construction of pJC-Amp, the *1169* promoter and *amp* gene were amplified from pJB-CAT by PCR. The PCR products were cloned by In-Fusion into SalI/NheI-digested pJC-CAT to create pJC-Amp. For construction of pMini-Tn7T-Kan, the *1169*^*P*^-*kan* fragment was amplified from pJB-Kan by PCR. The *1169*^*P*^-*kan* amplicon was cloned by In-Fusion into SalI-digested pMini-Tn7T-CAT to create pMini-Tn7T-Kan.

### Construction of pJC-amp::*pldA*-5′3′-CAT for targeted gene deletion

The *pldA* 5′ and 3′ flanking regions were amplified from Nine Mile (RSA439) genomic DNA by PCR. The PCR products were cloned by In-Fusion into BamHI/SalI-digested pJC-Amp to create pJC-Amp::*pldA*-5′3′. The *1169*^*P*^-*cat* fragment was amplified from pJB-CAT by PCR. The *1169*^*P*^-*cat* amplicon was cloned by In-Fusion into PstI-digested pJC-Amp::*pldA*-5′3′ to create the knock out vector pJC-Amp::*pldA*-5′3′-CAT.

### Construction of pMini-Tn7T-Kan::*pldA*comp for complementation

The *pldA* gene and its upstream promoter region was amplified from Nine Mile (RSA439) genomic DNA by PCR. The PCR product was cloned by In-Fusion into EcoRI-digested pMini-Tn7T-Kan to create pMini-Tn7T-Kan::*pldA* comp.

### *C. burnetii* gene deletion and complementation

Deletion of *pldA* was achieved as previously described using pJC-Amp::*pldA*-5′3′-CAT and 3 μg/ml chloramphenicol for antibiotic selection [39]. The mutant strain was cloned by picking colonies propagated on ACCM-2 agarose. Gene deletion was confirmed by PCR. *C. burnetii* Δ*pldA* was complemented with single copy pMini-Tn7T-Kan::*pldA*comp as previously described using 350 μg/ml kanamycin for antibiotic selection [16].

### Antibody generation and immunoblotting of *C. burnetii* lysates

Monospecific rabbit polyclonal antibody directed against *C. burnetii* PldA was generated using the PldA-specific synthetic peptide CRHIKRYDKKTKHY (Alpha Diagnostic International, San Antonio, TX). Cell lysates were prepared by boiling 2 × 10^8^ genomic equivalents (GE) of each *C. burnetii* strain in Laemmli sample buffer for 10 min. Proteins were separated by sodium dodecyl sulfate–polyacrylamide gel electrophoresis using a 4–20% gel (Bio-Rad), transferred to nitrocellulose, and probed with the anti-PldA antibody. Reacting proteins were detected using anti-rabbit IgG secondary antibodies conjugated to horseradish peroxidase (Thermo Scientific, Waltham, MA) and chemiluminescence using ECL Pico reagent (Thermo Scientific).

### Quantification of *C. burnetii* replication

Replication was quantified by qPCR (quantitative polymerase chain reaction) of *C. burnetii* GE using a primer and probe set specific to *C. burnetii dotA* [2]. ACCM-2 cultures were inoculated with 1 × 10^6^ GE. THP-1 monocytes were differentiated into macrophage-like cells with phorbol-12-myristate-13-acetate (PMA), then infected at a multiplicity of infection (MOI) of 0.2 [40]. Samples were taken immediately and 6 days post-inoculation/infection. Each sample was diluted in 150 μl phosphate-buffered saline (PBS) and boiled for 10 min prior to qPCR. Three independent experiments were performed in triplicate.

### Immunofluorescence and CCV analysis

THP-1 cells were seeded on coverslips in 24-well plates at a density of 1 × 10^5^ cells per well, stimulated with PMA for 1 day, then infected at an MOI of 10. At 4 days post-infection, cells were fixed for 30 min with 4% paraformaldehyde, then permeabilized and blocked with 0.1% Triton X-100 containing 1% bovine serum albumin. Cells were fluorescently stained for CD63 (mouse monoclonal antibody H5C6, BD Pharmingen) and *C. burnetii* (rabbit anti-Nine Mile phase II strain antibody). Alexa Fluor-488 goat anti-mouse and Alexa Fluor-647 goat anti-rabbit antibodies were from Life Technologies. For staining nuclei, Hoescht 33342 (ThermoFisher) was used and for visualization of cell borders, the filamentous actin stain, BODIPY 558/568 labeled phalloidin (Life Technologies) was used. Imaging was performed on a Zeiss LSM-710 confocal fluorescence microscope (Carl Zeiss). Fiji (Image J; National Institutes of Health, USA) was used for measuring areas of CCVs, where CD63 served as a CCV membrane marker. A minimum of 80 cells for each infection from 3 independent experiments were used for analysis.

### Isolation and analysis of lipid species

*E. coli* was grown to an optical density of 1, harvested at 4000×*g* for 20 min, then the pellet washed once with PBS (1.5 mM KH_2_PO_4_, 2.7 mM Na_2_HPO_4_-7H_2_O, 155 mM NaCl, [pH 7.2]). *C. burnetii* was grown for 4, 7, and 14 days, harvested at 20,000×*g* for 20 min, then the pellet washed once with PBS. Lipids were extracted by the method of Bligh and Dyer [41]. Dry weights were determined and the lipids spotted onto a Silica Gel 60 TLC plate (*E. coli,* 150 to 200 μg per lane; *C. burnetii,* 400 to 550 μg per lane). Lipids were separated using a solvent system containing chloroform, methanol, and glacial acetic acid (65:25:10, *v*/v). To visualize the lipids, TLC plates were sprayed with 10% sulfuric acid in ethanol and heated on a hot plate at 200 °C. Standards were obtained from the following suppliers and used in the indicated quantities: 18:1 Lyso-PE, 50 μg (Avanti Polar Lipids, Alabaster, AL); palmitoleic acid, 5 μg (Sigma-Aldrich, St. Louis, MO).

### Statistical analysis

Statistical analyses were performed using the unpaired Student *t* test and GraphPad Prism 6.0 software (La Jolla, CA).

## Additional files

Additional file 1: **Figure S1.** Schematic of allelic exchange procedure for generation of a *pldA* null mutant. (TIF 87 kb)

Additional file 2: **Table S1.** Bacterial strains and plasmids used in this study. (DOCX 17 kb)

Additional file 3: **Table S2.** Oligonucleotide primers used in this study. (DOCX 14 kb)

## Abbreviations

18–1 Lyso-PE: 18–1 lyso-phosphatidylethanolamine
ACCM-2: acidified citrate cysteine medium-2
*C. burnetii*: *Coxiella burnetii*
CCV: *Coxiella*-containing vacuole
CL: cardiolipin
*E. coli*: *Escherichia coli*
FFA: free fatty acid
GE: genomic equivalent
LB: Luria-Bertani
LCV: large cell variant
lyso-PL: lyso-phospholipid
MOI: multiplicity of infection
PBS: phosphate-buffered saline
PC: phosphatidylcholine
PCR: polymerase chain reaction
PE: phosphatidylethanolamine
PG: phosphatidylglycerol
PI: phosphatidylinositol
PldA: outer membrane phospholipase A
PMA: phorbol-12-myristate-13-acetate
PS: phosphatidylserine
qPCR: quantitative polymerase chain reaction
R_f_: retardation factor
SCV: small cell variant
TLC: thin layer chromatography
