# Supplementary figures and images for: A Coxiella burnetii phospholipase A homolog pldA is required for optimal growth in macrophages and developmental form lipid remodeling
Source: BMC Microbiol. 2018 Apr 16;18:33. doi: 10.1186/s12866-018-1181-0 (PMC5902883; doi:10.1186/s12866-018-1181-0)

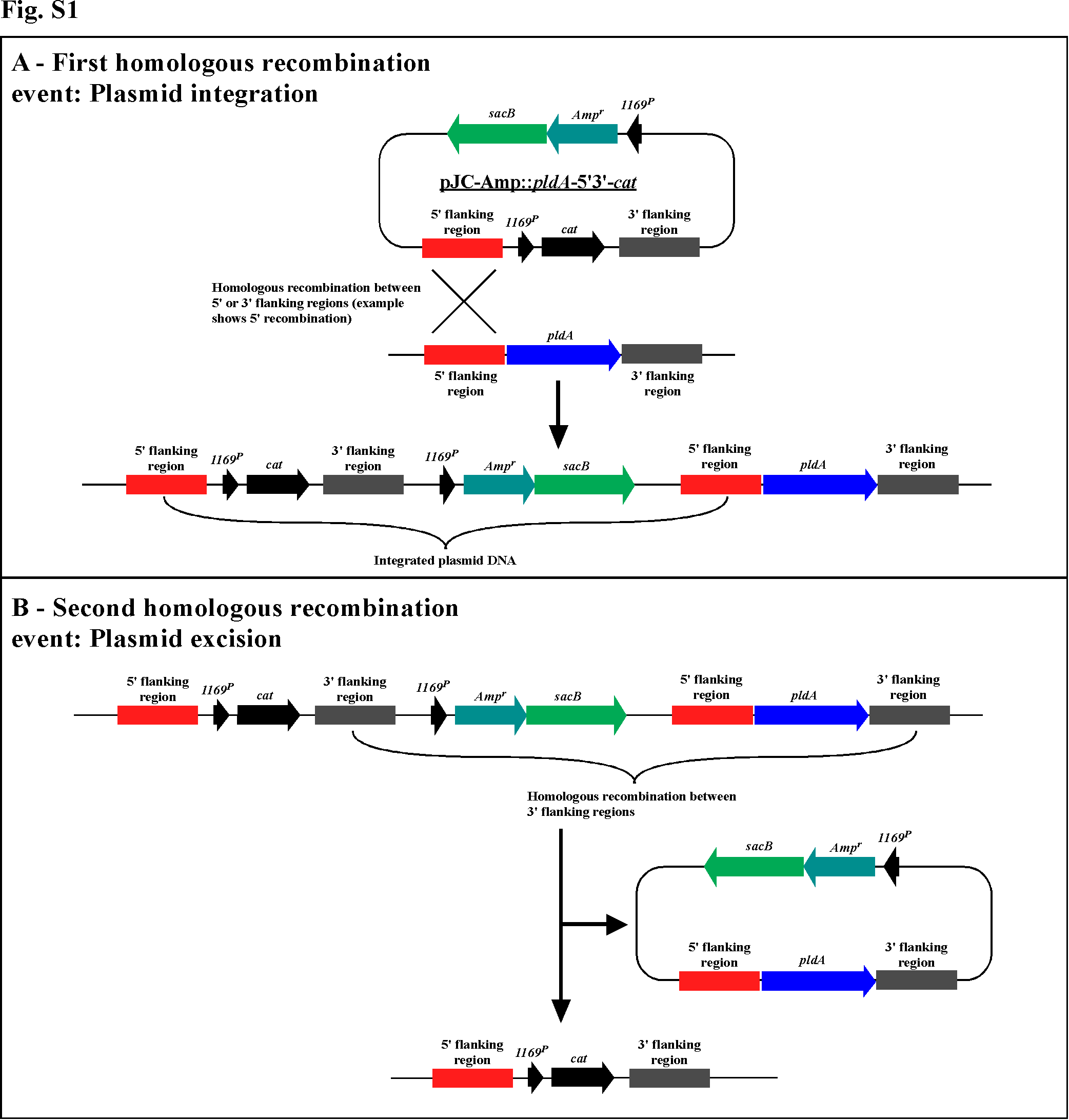

Supplement: Supplementary file 1 — Figure S1. Schematic of allelic exchange procedure for generation of a pldA null mutant. (TIF 87 kb) [file 12866_2018_1181_MOESM1_ESM.tif]
