# Supplementary material for: A Coxiella burnetii phospholipase A homolog pldA is required for optimal growth in macrophages and developmental form lipid remodeling
Source: BMC Microbiol. 2018 Apr 16;18:33. doi: 10.1186/s12866-018-1181-0 (PMC5902883; doi:10.1186/s12866-018-1181-0)
Supplement: Supplementary file 2 — Table S1. Bacterial strains and plasmids used in this study. (DOCX 17 kb) [file 12866_2018_1181_MOESM2_ESM.docx]

| Supplemental Table 1. Bacterial strains and plasmids used in this study | | |
| --- | --- | --- |
| Strain or plasmid | Genotype and/or phenotype^a^ | Source or reference |
| Strains |  |  |
| *C. burnetii* |  |  |
| Nine Mile, phase II (NMII) | Clone 4, RSA439 | (11) |
| NMII/Δ*pldA* | *pldA* deletion mutant; Cm^r^ | This study |
| NMII/Δ*pldA*comp | *pldA* deletion mutant complemented with *pldA^P^*-*pldA*; Cm^r^, Kan^r^ | This study |
| *E. coli* |  |  |
| Stellar | *F-*, *endA1*, *supE44*, *thi-1*, *recA1*, *relA1*, *gyrA96*, *phoA*, *Φ80d* *lacZ*Δ *M15*, Δ (*lacZYA* - *argF*) *U169*, Δ(*mrr* - *hsdRMS* - *mcrBC*), Δ*mcrA*, λ– | Clontech |
| W3110 | F-, λ- | *E. coli* Genetic Stock Center, Yale University |
| Plasmids | |  |
| pJC-CAT | pJC84 containing *cat* driven by *1169^P^*; Cm^r^ | (40) |
| pJB-CAT | pJB2581 containing *cat* driven by *1169^P^*; Cm^r^, Amp^r^ | (38) |
| pJB-Kan | pJB2581 containing *kan* driven by *1169^P^*; Kan^r^, Amp^r^ | (38) |
| pJC-Amp | pJC84 containing *amp* driven by *1169^P^*; Amp^r^ | This study |
| pJC-Amp::*pldA*-5′3′ | 5' and 3' flanking DNA from *pldA* cloned into pJC-Amp; Amp^r^ | This study |
| pJC-Amp::*pldA*-5′3′-CAT | *1169^P^*-*cat* cassette cloned into pJC-Amp::*pldA*-5'3'; Cm^r^, Amp^r^ | This study |
| pTnS2::*1169^P^*-*tnsABCD* | *1169^P^* cloned into pTnS2; Amp^r^, R6K ori | (16) |
| pMini-Tn7T-CAT | *1169^P^*-*cat* cloned into pUC18R6K-mini-Tn7T; Cm^r^, Amp^r^, R6K ori | (16) |
| pMini-Tn7T-Kan | *1169^P^*-*kan* cloned into pUC18R6K-mini-Tn7T; Kan^r^, Amp^r^, R6K ori | This study |
| pMini-Tn7T-Kan::*pldA*comp | *pldA*comp fragment cloned into pMiniTn7T-Kan; Kan^r^, Amp^r^ | This study |
| ^a^ Cm^r^, chloramphenicol resistance; Amp^r^, ampicillin resistance; Kan^r^, kanamycin resistance. | | |
