# Supplementary material for: A Coxiella burnetii phospholipase A homolog pldA is required for optimal growth in macrophages and developmental form lipid remodeling
Source: BMC Microbiol. 2018 Apr 16;18:33. doi: 10.1186/s12866-018-1181-0 (PMC5902883; doi:10.1186/s12866-018-1181-0)
Supplement: Supplementary file 3 — Table S2. Oligonucleotide primers used in this study. (DOCX 14 kb) [file 12866_2018_1181_MOESM3_ESM.docx]

| Supplemental Table 2. Oligonucleotide primers used in this study | |
| --- | --- |
| Primer | Sequence (5*'* to 3*'*) |
| Primers for construction of pJC-Amp | |
| AmpforpJC-F | CATGAAGGAGGGCCCTTATGAGTATTCAACATTTCCGTGTC |
| AmpforpJC-R | CATGTCTCCTGCTAGCTTACCAATGCTTAATCAGTGAG |
| *1169^P^*forpJC-F | ATCCTCTAGAGTCGACAAACAGGTTCTCTAATTAATCCG |
| *1169^P^*forpJC-R | AAGGGCCCTCCTTCATGAAGGATTAATGTCATTATTTATTTATGGGGTATG |
| Primers for construction of pMini-Tn7T-Kan | |
| *1169^P^*-KAN-Tn7-F | TATCGATACCGTCGACATGGCTTCGTTTCGCAGCG |
| Kan-Tn7-R | GGGGTTCGAGGTCGACTTATCAGAAGAACTCGTCAAGAAGGCG |
| Primers for construction of pJC-Amp::*pldA*-5′3′-CAT | |
| CBU0489-5′-F | CGGTACCCGGGGATCCTTCGTAGCGCTTGAGAAGGCGG |
| CBU0489-5′-R | CACCCTGCAGCGACGCGAGCGTCGAGGGAAGGATAGTATCAAAATTCTTC |
| CBU0489-3′-F | CGTCGCTGCAGGGTGCGCATGTACGTCGACTTCGCTCGAGATCACAC |
| CBU0489-3-′R | GAACCTGTTTGTCGACGGGCGCTCAACCACAAACTTGG |
| *1169^P^*-CAT-PstI-KO-F | CGACGCTCGCGTCGCTGCAGATGGCTTCGTTTCGCAGCGAACTTGG |
| *1169^P^*-CAT-PstI-KO-R | CGTACATGCGCACCCTGCAGTTACGCCCCGCCCTGCCACTC |
| Primers for confirming gene deletion | |
| CBU0489-KO-F | CGGAAAAGCCATCAGAAAAGTG |
| CBU0489-KO-R | AATCCAATTGCTGATAATAATTCC |
| Primers for construction of pMini-Tn7T-Kan::*pldA*comp | |
| CBU0489comp-F | CGGGCTGCAGGAATTCAAACAAATGGGTGCCTATTAG |
| CBU0489comp-R | GCTTCTCGAGGAATTCCTAAATCCAATTGCTGATAATAATTCC |
| Locations of restriction enzyme sites are underlined. | |
